# Systemic inflammatory response in robot-assisted and laparoscopic surgery for colon cancer (SIRIRALS): study protocol of a randomized controlled trial

**DOI:** 10.1186/s12893-021-01355-4

**Published:** 2021-10-11

**Authors:** Pedja Cuk, Andreas Kristian Pedersen, Kate Lykke Lambertsen, Christian Backer Mogensen, Michael Festersen Nielsen, Per Helligsø, Ismail Gögenur, Mark Bremholm Ellebæk

**Affiliations:** 1 Surgical Department, Hospital Sønderjylland, University Hospital of Southern Denmark, Kresten Philipsens vej 15, 6200 Aabenraa, Denmark; 2 OPEN, Odense Patient Data Explorative Network, Odense University Hospital, Odense, Denmark; 3 Institute of Regional Health Research, University of Southern Denmark, Odense, Denmark; 4 Department of Neurobiology Research, Institute of Molecular Medicine, University of Southern Denmark, Odense, Denmark; 5 Department of Neurology, Odense University Hospital, Odense, Denmark; 6 BRIDGE, Brain Research - Inter-Disciplinary Guided Excellence, Department of Clinical Research, University of Southern Denmark, Odense, Denmark; 7 Surgical Department, Center for Surgical Science, Zealand University Hospital, Roskilde, Denmark; 8 Surgical Research Unit, Odense University Hospital, Odense, Denmark; 9 University of Southern Denmark, Odense, Denmark

**Keywords:** Inflammatory surgical stress response, Minimally invasive surgery, Colon cancer, Robot-assisted surgery, Laparoscopic surgery

## Abstract

**Background:**

Robot-assisted surgery is being increasingly adopted in treating colorectal cancer, and the transition from laparoscopic surgery to robot-assisted surgery is a trend. The evidence of the benefits of robot-assisted surgery is sparse. However, findings are associated with improved patient-related outcomes and overall morbidity rates compared to laparoscopic surgery. This induction is unclear, considering both surgical modalities are characterized as minimally invasive. This study aims to evaluate the systemic and peritoneal inflammatory stress response induced by robot-assisted surgery compared with laparoscopic surgery for elective colon cancer resections in a prospective, randomized controlled clinical trial.

**Methods:**

This study is a single-centre randomized controlled superiority trial with 50 colon cancer participants. The primary endpoint is the level of systemic inflammatory response expressed as serum C-reactive protein (CRP) and interleukin 6 (IL-6) levels between postoperative days one and three. Secondary endpoints include (i) levels of systemic inflammation in serum expressed by a panel of inflammatory and pro-inflammatory cytokines measured during the first three postoperative days, (ii) postoperative surgical and medical complications (30 days) according to Clavien-Dindo classification and Comprehensive Complication Index, (iii) intraoperative blood loss, (iv) conversion rate to open surgery, (v) length of surgery, (vi) operative time, (vii) the number of harvested lymph nodes, and (viii) length of hospital stay. The exploratory endpoints are (i) levels of peritoneal inflammatory response in peritoneal fluid expressed by inflammatory and pro-inflammatory cytokines between postoperative day one and three, (ii) patient-reported health-related quality of recovery-15 (QoR-15), (iii) 30 days mortality rate, (iv) heart rate variability and (v) gene transcript (mRNA) analysis.

**Discussion:**

To our knowledge, this is the first clinical randomized controlled trial to clarify the inflammatory stress response induced by robot-assisted or laparoscopic surgery for colon cancer resections.

*Trial registration* This trial is registered at Clinicaltrials.gov (Identifier: NCT04687384) on December, 29, 2020, Regional committee on health research ethics, Region of Southern Denmark (N75709) and Data Protection Agency, Hospital Sønderjylland, University Hospital of Southern Denmark (N20/46179).

**Supplementary Information:**

The online version contains supplementary material available at 10.1186/s12893-021-01355-4.

## Background

Laparoscopic colon surgery is an implemented and well-established surgical procedure for colon malignancies worldwide. The method was introduced in the 1990s and is associated with fewer wound-related complications, less pain, and similar pathological specimen quality compared to open colonic surgery [1–6]. However, the disadvantages of laparoscopic colon surgery include a prolonged learning curve, suboptimal ergonomics, tremor and camera position [7–9].

Within the last 20 years, robot-assisted colon surgery has become more widespread among several surgical specialities. To date, no randomized controlled trials comparing the systemic or peritoneal inflammatory response in colon cancer surgery performed by robot-assisted or laparoscopic methods has been published. A decreased systemic inflammatory response is associated with a lower risk of surgical complications and improved long-term oncological outcomes [10]. This response is due to a cascade of neuroendocrine, metabolic and immunological factors. In the systemic acute inflammatory reaction, a release of pro-inflammatory cytokines (IL-1β, IL-6 and TNF) occurs as part of wound healing by producing acute phase reactants (C-reactive protein (CRP), fibrinogen and complement C3) as a reaction to the initial surgical trauma [11, 12]. High concentrations of pro-inflammatory cytokines (predominantly IL-6) and lack of compensatory expression of anti-inflammatory cytokines may cause systemic inflammatory response syndrome (SIRS). There is a direct correlation between high concentrations of pro-inflammatory cytokines and an increased mortality rate [11, 13]. Non-randomized clinical trials have demonstrated that robot-assisted colorectal surgery has positive effects on faster establishment of bowel function and shorter hospital stay than laparoscopic surgery [14–17]. One study reported that robot-assisted surgery contributes to reduced surgical trauma due to better tissue exposure because of 3D-vision, wristed instruments and tremor reduction minimizing the inflammatory stress response, compared to the laparoscopic method [18].

This study aims to evaluate the systemic and peritoneal inflammatory stress response induced by robot-assisted surgery compared with laparoscopic surgery for elective colon cancer resections in a prospective, randomized controlled clinical trial.

## Methods/design

### Study design

A single-institution double-blinded randomized controlled trial was conducted at the tertiary care hospital (Surgical Department, Hospital Sønderjylland, University Hospital of Southern Denmark, Denmark) with participants requiring colon surgery for malignancy. The study protocol adheres to the guidelines determined in Standard Protocol Items: Recommendations for Interventional Trials (SPIRIT) [19].

#### Inclusion criteria

- Elective robot-assisted or laparoscopic surgery for right-sided, left-sided and sigmoid colon cancer.
- Age ≥ 18.
- ASA-score ≤ 3.
- Tumor-stage (T1–T4a).
- Endoscopic suspected colon cancer.
- Histologically verified adenocarcinoma, signet ring cell carcinoma, undifferentiated cancer, medullary carcinoma or another malignant tumour type originating from the colon.
- Patients must give written informed consent.
- Patients must be able to understand Danish.

#### Exclusion criteria

- Carcinoma of the transverse colon or synchronous colorectal cancer.
- Previous history of any colon resection.
- Previous open major abdominal surgery except for open appendectomy and cholecystectomy.
- Pregnancy.
- Metastatic disease.
- History of psychiatric or addictive disorder that would prevent the patient from participating in the trial.
- Emergency colon surgery.
- Co-existing inflammatory bowel disease.
- Co-existing immunological disease requiring ingestion of systemic immunomodulatory drugs (DMARD—disease-modifying anti-rheumatic drugs) - uploaded as Additional file 1, corticosteroids and/or biologic disease-modifying anti-rheumatic drugs.
- Daily consumption of NSAID drugs.

### Interventions

#### Surgical procedures

Patients will receive preoperatively the prophylactic antibiotics Piperacillin/Tazobactam 4 g + 0.5 g and Metronidazole 1.5 g, compression stockings and antithrombotic drugs (Dalteparin 5000 IU), urinary catheter, and a nasogastric tube. No mechanical bowel preparation will be used before surgery. According to oncological principles, a dedicated team of certified colorectal and robotic surgeons will perform all surgical procedures in an institution performing > 200 malignant colon resections yearly. Since the implementation of robot-assisted colorectal surgery in our institution in 2017, the operating surgeons' caseloads have surpassed the learning curve of 25–45 cases [20–23]. In addition, in our institution, laparoscopic colorectal resections have been performed since 2005, and the operating surgeons are familiar with these surgical techniques, surpassing the learning curve of approximately 50 cases [24]. Surgical procedures will be performed either laparoscopically or entirely robot-assisted. The robot-assisted procedures will be performed using a da Vinci Xi system (Intuitive Surgical, Sunnyvale, California, US).

#### Right-sided colectomy

In right-sided and extended right-sided colectomy, by consensus within our institution, the approach is lateral-to-medial. The transverse colon is dissected medial to lateral, proceeded by entering into the lesser sac. The gastrocolic ligament is divided, the hepatic flexure is mobilized, Toldt’s fascia is incised, and attachments to the lateral abdominal wall are dissected. The cecum is retracted cranially to expose the ileocolic pedicle. The dissection continues medially until the descending part of the duodenum and the pancreatic head are located where the gastrocolic trunk runs into the superior mesenteric vein. The ileocolic pedicle is isolated, clipped and divided individually with a sealer device at its origin. The dissection continues above the duodenum. The right colic artery and the right branch of the middle colic artery are identified and divided with a sealer device at the origin. The ileal mesentery is divided, and mobility is ensured to obtain a tension-free anastomosis. The terminal ileum and transverse colon are divided intracorporeally with a laparoscopic stapler. The specimen is extracted through a right-sided upper horizontal transverse muscle splitting incision. An extra-corporeal, hand-sewn, end-to-end, single layer, seromuscular tension-free anastomoses will be performed in both surgical modalities.

#### Left-sided colectomy

In left-sided colectomy, the approach will be lateral-to-medial. Peritoneal attachments of the lateral abdominal wall will be incised corresponding to Toldt’s fascia. The ureter is identified. The inferior mesenteric artery and vein are identified at the level of the pancreas, then clipped and divided separately with a sealer device. The dissection will continue proximally upwards toward the inferior border of the pancreas and distally to the sacral promontory. The mesocolon will be mobilized by preserving the hypogastric autonomic nerves. The gastrocolic ligament is divided, and the lesser sac is entered. The dissection is continued by division of the splenocolic ligament, and the splenic flexure is mobilized to ensure a tension-free anastomosis. The transverse colon and rectum are transected intracorporeally with a laparoscopic stapler. A muscle splitting incision is performed in the left iliac fossa, and the specimen is extracted. An extra-corporeal end-to-end hand-sewn or stapled, tension-free seromuscular anastomosis will be performed depending on tumor location and bowel length, as we prefer to perform hand-sewn anastomosis.

#### Sigmoid colectomy

The procedure is initiated by the lateral mobilization of Toldt’s fascia, including mobilization of the splenic flexure. The left ureter is identified after the completion of lateral dissection. The origin of the inferior mesenteric artery and superior rectal artery is identified. The inferior mesenteric artery and vein are clipped and divided with a sealer device. The left colon is dissected posteriorly, the pancreatic tail is identified, the greater omentum is incised to enter the lesser sac. The greater omentum is dissected medial to lateral, the splenocolic ligament is divided, and the splenic flexure is mobilized to ensure a tension-free colorectal anastomosis. The colon is divided intracorporeally, proximally and distally with a laparoscopic stapler. A muscle splitting incision is performed in the left iliac fossa, and the specimen is extracted. A stapled end-to-end colorectal anastomosis is performed. An intra-operative air leak test tests the anastomosis.

### Peritoneal microdialysis

Patients will have a 61 high cut-off microdialysis catheter (CMA 61; CMA Microdialysis AB, Stockholm®, Sweden) inserted intraperitoneally at the end of surgery. The catheter will be perfused with an isotonic perfusion fluid (Dextran) used for microdialysis, with a flow rate of 0.3 μl/min. The catheter membrane allows large molecules to diffuse into the perfusion fluid due to its permeability as it mimics a capillary [25]. Approximately 1.5 ml of intraperitoneal fluid will be collected from the abdominal cavity once daily for 3 days postoperatively.

### Postoperative management

The nasogastric tube will be removed after the cessation of anaesthesia. Both robot-assisted and laparoscopic colon surgery are categorized as minimally invasive and follow the principles of enhanced recovery after surgery (ERAS) to minimize the risk of postoperative complications, morbidity, and hospitalization time [26] and Additional file 2. Patients will be encouraged to mobilize through daily consultations with a physiotherapist. Antithrombotic treatment will continue four weeks postoperatively.

### Anesthesia and postoperative analgesic management

The operation is performed under general anaesthesia in both surgical groups and will consist of:

#### Preoperative medication

The patients will be medicated with Paracetamol (1 g × 4) and Ondansetron (4 mg).

#### Maintenance of general anaesthesia

- Propofol (1–3 mg/kg) initially followed by a continuous dose of 5 mg/kg/hour.
- Sufentanil (0.5 μg/kg) initially followed by a continuous dose of 1,5 μg/kg.
- Rocuronium (0.6 mg/kg) initially followed by a continuous dose of 0.3–0.6 mg/kg.
- Oxycodone (0.1 mg/kg) 30 minutes before the cessation of the procedure.
- Rocuronium if train-of-four is 0–1.
- Remifentanil (0.15 μg/kg).

Dexamethasone and NSAID will be omitted. The total dose of anaesthetics is continuously recorded.

#### Postoperative management

- Paracetamol 1 g × 4.
- Oxycontin 5–10 mg × 2.
- Oxynorm 5–10 mg × 2 (pro necessaire).
- Ondansetron 4 mg (pro necessaire).

### Outcomes

**Primary endpoint**:

- Levels of systemic inflammatory response expressed by serum C-reactive protein (CRP) and interleukin 6 (IL-6) measured postoperatively day one to day three
  **Secondary endpoints** 
- Levels of systemic inflammation in serum expressed by: Eotaxin, Eotaxin-3, granulocyte–macrophage colony-stimulating factor (GM-CSF), interferon-gamma (IFN-γ), interleukin 1 alpha (IL-1α), interleukin 1 beta (IL-1β), interleukin 2 (IL-2), interleukin 4 (IL-4), interleukin 5 (IL-5), interleukin 7 (IL-7), interleukin 8 (IL-8), interleukin 10 (IL-10), interleukin 12 (IL-12), interleukin 23 (IL-23), interleukin 13 (IL-13), interleukin 15 (IL-15), interleukin 16 (IL-16), interleukin 17a (IL-17A), interferon gamma-induced protein 10 (IP-10), monocyte chemoattractant protein 1 (MCP-1), monocyte chemoattractant protein 4 (MCP-4), macrophage derived chemokine (MDC), macrophage inflammatory protein 1 alpha (MIP-1α), macrophage inflammatory protein 1 beta (MIP-1β), thymus and activation regulated chemokine (TARC), tumor necrosis factor alpha (TNF-α), tumor necrosis factor alpha (TNF-β) lymphotoxin alpha (LT-α), vascular endothelial growth factor A (VEGF-A) and interleukin 1 receptor antagonist (IL-1RA) measured on the first three postoperative days.
- Postoperative surgical and medical complications (30 days) according to Clavien-Dindo classification [27] and Comprehensive Complication Index [28].
- Postoperative dynamic pain registration (visual analog scale (VAS)) at rest twice daily (morning and evening) and registration of opioid consumption in medical charts.
- Intraoperative blood loss (mL).
- Conversion rate to open surgery.
- Total surgical duration (minutes).
- Total anaesthesia duration (minutes).
- Lymph node yield.
- Length of hospital stay (hours/days).
- Time to passage of flatus (hours).
- Time to passage of stool (hours/days).

#### Exploratory endpoints

- Levels of peritoneal inflammatory response in peritoneal fluid expressed by: Eotaxin, Eotaxin-3, GM-CSF, IFN-γ, IL-1α, IL-1β, IL-2, IL-4, IL-5, IL-6, IL-7, IL-8, IL-8 (HA), IL-10, IL-12, IL-13, IL-15, IL-16, IL-17A, IP-10, MCP-1, MCP-4, MDC, MIP-1α, MIP-1β, TARC, TNF, LT-α, VEGF-A, as well as IL-1RA and CRP measured on the first three postoperative days.
- Patient-reported health-related quality of recovery-15 (QoR-15) [29].
- 30 days mortality rate.
- Heart rate variability.
- Gene transcript (mRNA) analysis.
- Comparison of the systemic and peritoneal inflammatory response in relation to the risk of cancer recurrence 3 years postoperatively.

### Participant timeline, data collection and follow-up

**Fig. 1:**
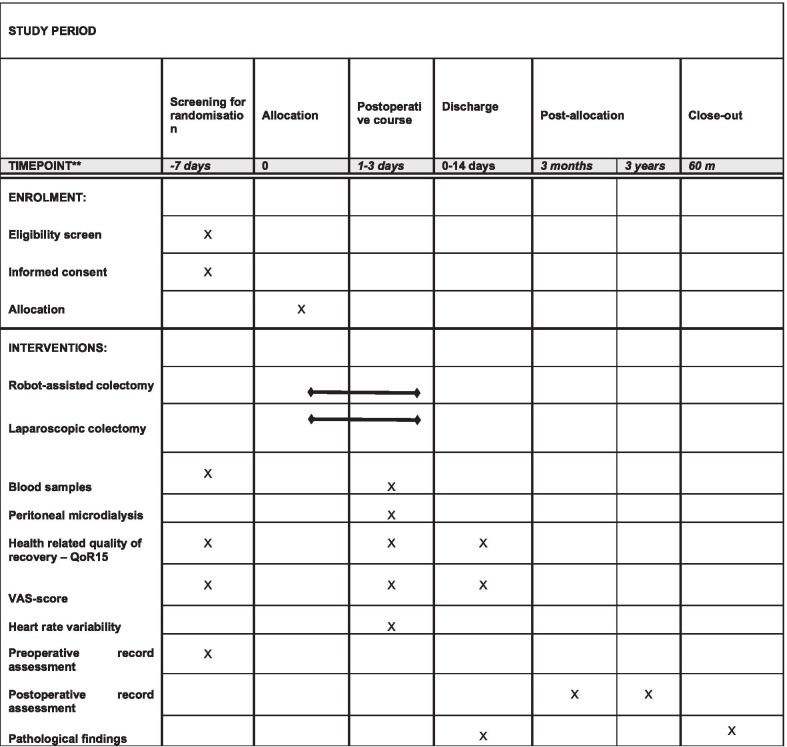
Schedule of Enrolment, Interventions, and Assessments (SPIRIT figure)

**Fig. 2:**
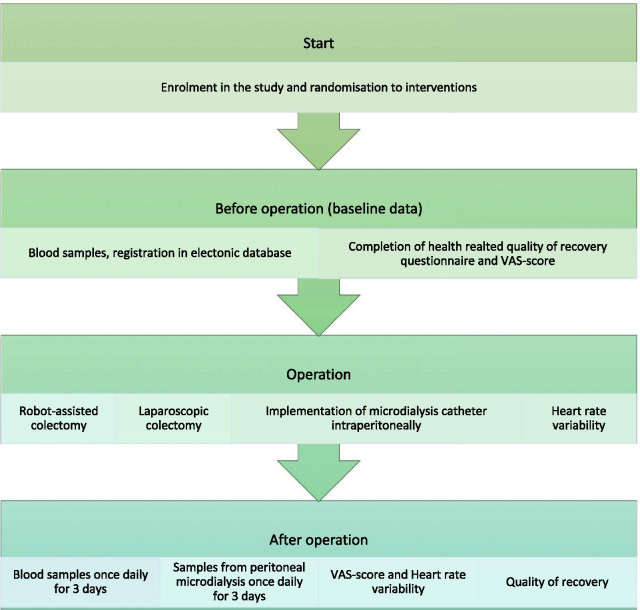
Participant timeline

The preoperative assessment will consist of information concerning baseline data (age, gender, body-mass index (BMI), ASA-score, Charlson Comorbidity Index (CCI), WHO performance status, date of diagnosis, type of planned operation, date of randomization and operation (Figs. 1 and 2). Patients will be preoperatively asked to fill out the QoR-15 questionnaire at baseline and from the first until the third postoperative day [29]. A full recovery status (QoR-15) will be collected 14 days postoperatively.

Short-term results and long term follow up of the study (peritoneal inflammatory response in comparison with systemic inflammatory stress response and the 3-year cancer recurrence) will be published separately. All sample data will be entered into an electronic database program (REDCap ®) hosted by the Open Patient data Explorative Network (OPEN), available only to project owners, ensuring that anonymity is maintained and data security is respected. Consent statements from participants in the study will be stored in accordance with GDPR regulations [30]. Ethical principles for medical research will follow the Helsinki declaration [31]. Blood samples and peritoneal fluid will be stored at a secure local study site. Once all patients are included in the study, the samples will be transferred to the Department of Neurobiology, the University of Southern Denmark, for laboratory analysis. Gene transcription analysis (mRNA) will be completed at the Zealand University Hospital, Køge, Denmark.

### Sample size

The Monte Carlo method calculated the power of the mixed effect model used for the log-normal distributed outcome of C-reactive protein (CRP) levels. The sample size calculation was based on the primary outcome (the systemic inflammatory postoperative response expressed by pro-inflammatory and anti-inflammatory cytokines). It was assumed that the CRP level in the group of patients undergoing laparoscopic surgery for colorectal cancer would be 19% higher than patients with robot-assisted surgery, with an interpersonal variation of 0.63 and an intrapersonal variation of 0.34 for the logarithmic transformed CRP. The mixed-effect model was adjusted for type of operation and time. The assumption of CRP among elective malignant colonic resections was based on postoperative measurements on days 1–3 from an observational data set of 298 patients undergoing surgery for colorectal cancer using robot-assisted or laparoscopic methods from 2017 to 2019 at our surgical department, Hospital Sønderjylland, University Hospital of Southern Denmark  [17]. To obtain a power of 86% and an alpha level of 0.05 for a two-sided p-value, we aimed to include a minimum of 42 patients.

### Recruitment, randomization and blinding

**Fig. 3:**
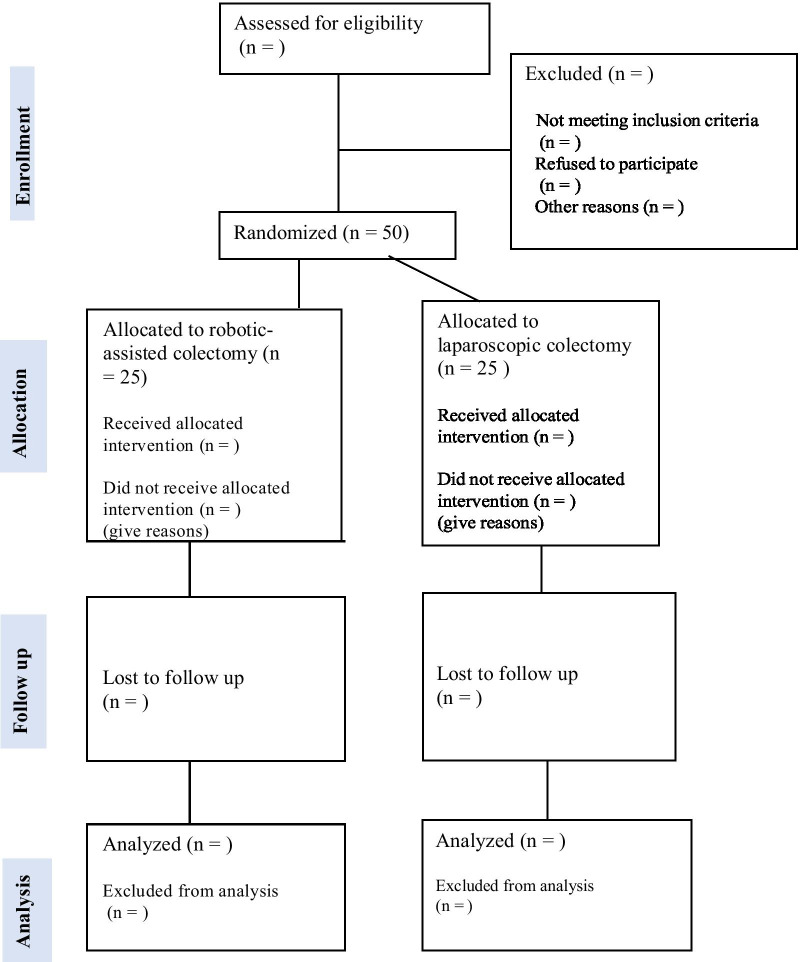
CONSORT flow diagram

A total of 50 patients (25 in each arm) will be recruited for randomization in the period 1st August 2021–1st September 2022 at the Surgical Department, Hospital Sønderjylland, University Hospital of Southern Denmark. The screening of potential candidates eligible for randomization will occur at a multidisciplinary colorectal cancer conference held twice weekly at the study site. Patients will be randomized according to transparent reporting of trials (CONSORT) on a 1:1 basis to either robot-assisted or laparoscopic surgery (Fig. 3). A computer-generated randomization sequence program (REDCap®) managed by the project owner will allocate patients to robot-assisted or laparoscopic colectomies. The program is provided with personal identification numbers that will be utilized, whereby data can be pseudo-anonymized. Subsequently, information material about the trial will be provided, including a health quality questionnaire. The study participants and data assessor will be blinded. The randomization will require the following information:

- Age and sex
- Confirmation that inclusion and exclusion criteria are met
- Confirmation of informed consent
- Planned date of operation
- Type of operation

### Statistics

Descriptive statistics will be utilized to check for exchangeability between the two groups according to baseline variables. Categorical variables will be analyzed with a Chi-square or Fischer’s exact test depending on Cochran’s rule. Continuous and discrete variables will be analyzed with an unpaired t-test or Wilcoxon rank-sum test depending on the distribution of the variables. The primary, secondary and exploratory endpoints will also be compared using descriptive statistics. For continuous and discrete variables, repeated measurement ANOVA or Kruskal–Wallis rank-sum tests will be used to compare these variables across timepoints stratified according to the operation method. The normality and heterogeneity of variance assumptions for the t-test and the ANOVA will be graphically assessed using quantile–quantile plots. A mixed-effect model will be utilized to analyze the degree of systemic and peritoneal inflammatory response because of repeated measurements. Logistic regression will be utilized to analyze perioperative complications, 30-day mortality, surgical and medical complications. Odds ratios with 95% confidence intervals will be reported. A poisson or linear regression will be used for time from treatment start until end, and the Quality of Recovery questionnaire (QoR-15 score). Quantile–quantile plots will be used to check if the deviance and the Pearson residuals are normally distributed, and a scatterplot of the deviance and Pearson residuals will check the closeness of the model to our data. If the residuals for the mixed-effect model are not normally distributed, a log transformation of the outcome may be necessary. If the residuals are still not normal distributed, a mixed-effect gamma model will be performed. If these residuals are not normally distributed, logistic regression with another type of link function will be utilized. If deviance residuals are not normally distributed and are not close to zero for the poisson regression, a negative binomial regression or linear regression with bootstrapped confidence intervals will be utilized. If adjustments in the mixed-effect model are necessary, a univariate analysis with only the exposure variable will be included as a sensitivity analysis. If the negative binomial regression is utilized, a poisson regression will be added as a supplementary analysis. No subgroups analysis will be a part of this project. All regression analyses will follow the one-in-ten rule to avoid overfitting. No interim analyses will be performed as no similar prospective randomized clinical trial has previously been performed, so completion of this study is of great importance.

### Ethics

Patients included in this study will undergo the same surgical procedures with both surgical modalities available before the study was established. Both surgical procedures are used internationally, and it is expected that the majority of the potential complications are well-known and not directly related to the study. Intraperitoneal microdialysis has been previously described without presenting major complications as a method [32–36]. The patients may feel discomfort around the microdialysis catheter. Compared with the pain around the incisions they receive during the surgical procedure, the discomfort of discontinuation of the microdialysis catheter can be considered minimal. Possible side effects of catheter administration may be subcutaneous infections. The catheter will be inspected daily for signs of infection such as purulent production, redness and pain. The catheter is equipped with a pump that can be fixed to the patient’s clothes. Wearing the pump can be associated with discomfort. Patients may experience a slight transient pain associated with catheter removal. If unexpected complications occur during the trial, this will be interrupted and reported as an unintended event. In case of significant changes in the study protocol, an updated version will be available and distributed to the regional committee of health-related ethics and clinicaltrials.gov. The study is registered with the regional committee on health research ethics, Region of Southern Denmark (N75709), Data Protection Agency, Hospital Sønderjylland, University Hospital of Southern Denmark (N20/46179) and clinicaltrials.gov (NCT04687384). A national insurance covers participants in case of unexpected complications that may occur during the trial. No financial resources will be provided to the trial participants. The principal investigator and collaborators have no financial interest in the trial. The final data set will be available from the project owner and data assessor. On request, data can be shared in an anonymized form if a data processor agreement is obtained. The principal investigator is responsible for monitoring the study, and unexpected complications must be reported to the regional committee of health-related ethics. The first author will regularly monitor the study’s process and ensure an action plan is implemented if problematic conditions arise.

### Dissemination policy

The study results will be published in a scientific international peer-reviewed journal and relevant conferences in anonymized form. Results will be available to participants, healthcare professionals, the public, and other relevant groups. In addition, the study protocol will be publicly accessible.

## Discussion

To our knowledge, this study is the first randomized controlled trial to investigate the surgical stress response induced by elective malignant colon surgery in robot-assisted versus laparoscopic surgery. In benign and malignant abdominal surgery, the immunological stress response has been studied in other closely related surgical specialities such as gynaecology and urology. However, most studies investigate the surgical stress response in robot-assisted versus open surgery, which is not comparable with this study as there is significantly more trauma with open surgery compared to minimally invasive surgery [10, 37–40]. It is a common conclusion in many studies that robot-assisted surgery initiates a lower stress response. However, open surgery for elective malignant colon cancer disease is not contemporary and has largely been replaced by minimally invasive surgery. The rationale for this study is the presumption that robot-assisted surgery induces a lower stress response as a consequence of minimal tissue traumatization compared to laparoscopic surgery. Minimal traumatization is possible due to the precise three-dimensional camera vision and improved tactile sense. In addition, the surgical trauma causes increased activation of macrophages intraperitoneally, thereby stimulating an increase in cytokine levels (predominantly interleukin 1 (IL-1), interleukin 6 (IL-6) and tumour necrosis factor (TNF-α)) [41].

Furthermore, the study will examine the systemic stress response by peritoneal microdialysis and elucidate the intraperitoneal stress response presumed to be higher with surgical trauma. Systematic reviews and meta-analyses on colorectal robot-assisted versus laparoscopic surgery have confirmed robot-assisted surgery has lower complication and conversion rates, faster hospital discharge and restoration of bowel function [5, 14, 16, 42, 43]. However, the studies are primarily based on observational study designs, where the potential for selection bias and confounding factors could have influenced the conclusion. By conducting a prospective randomized study, which accounts for preoperative influencing factors, it is possible to obtain a uniform patient population, where the systemic and peritoneal stress response induced by the two surgical methods are comparable. The study will also evaluate the clinical short-term outcomes and quality of life reflected in the lower stress response by robot-assisted surgery.

## Supplementary Information

**Additional file 1.** List of most common DMARD drugs.

**Additional file 2.** ERAS (enhanced recovery after surgery) protocol.

## Data Availability

Not applicable.

## Abbreviations

ASA: American Society of Anesthesiologists physical status
DMARD: Disease-modifying anti-rheumatic drugs
NSAID: Non-Steroidal Anti-Inflammatory Drugs
IU: International unit
ERAS: Enhanced Recovery After Surgery
CRP: C-reactive protein
IL: Interleukin
GM-CSF: Granulocyte–Macrophage Colony-Stimulating factor
IFN: Interferon
IP: Interferon Gamma-induced Protein
MCP: Monocyte Chemoattractant Protein
MDC: Macrophage Derived Chemokine
MIP: Macrophage Inflammatory Protein
TARC: Thymus And Activation Regulated Chemokine
TNF-α: Tumor Necrosis Factor alpha
LT: Lymphotoxin
VEGF: Vascular Endothelial Growth Factor
IL-1RA: Interleukin 1 receptor antagonist
VAS: Visual Analog Scale
QoR15: Patient reported health related quality of recovery-15
mRNA: Messenger RNA
BMI: Body Mass Index
CCI: Charlson Comorbidity Index
WHO: World Health Organization
GDPR: General data protection regulation
t-test: Student’s t-distribution
ANOVA: Analysis of Variance
