# Supplementary material for: Systemic inflammatory response in robot-assisted and laparoscopic surgery for colon cancer (SIRIRALS): study protocol of a randomized controlled trial
Source: BMC Surg. 2021 Oct 11;21:363. doi: 10.1186/s12893-021-01355-4 (PMC8507379; doi:10.1186/s12893-021-01355-4)
Supplement: Supplementary file 1 — Additional file 1. List of most common DMARD drugs. [file 12893_2021_1355_MOESM1_ESM.docx]

**Additional file 1: List of most common DMARD drugs**

- Methotrexate (Methotrexat, Ebetrex®, Emthexate®, Injexate®, Metex®, Trexan®)
- Cyclophosphamid (Sendoxan®)
- Sulfasalazine (Salazopyrin®)
- Ciclosporin (Sandimmun®, Adport®, Advagraf®, Ciqorin®, Envarsus®, Prograf®, Modigraf®)
- Leflunomide (Arava®)
- Azathioprine (Azathioprin, Imurel®)
- Mycophenolic acid (Cellcept®, Mycophenolatmofetil, Mycophenolsyre, Myfenax, Myfortic®)
- Chloroquine (Plaquenil®)
