# Supplementary material for: Systemic inflammatory response in robot-assisted and laparoscopic surgery for colon cancer (SIRIRALS): study protocol of a randomized controlled trial
Source: BMC Surg. 2021 Oct 11;21:363. doi: 10.1186/s12893-021-01355-4 (PMC8507379; doi:10.1186/s12893-021-01355-4)
Supplement: Supplementary file 2 — Additional file 2. ERAS (enhanced recovery after surgery) protocol. [file 12893_2021_1355_MOESM2_ESM.docx]

**Additional file 2: ERAS (enhanced recovery after surgery) protocol**

**ERAS (enhanced recovery after surgery) protocol of colon cancer surgery performed at the surgical department, University Hospital of Southern Jutland, Denmark**

**Preoperative interventions**

1. We assessed the operability at a multidisciplinary conference including a colorectal surgeon, oncologist, radiologist, pathologist, and nurse. The patient is informed about the course of the surgical procedure, risks, and complications in the outpatient clinic.
2. Screening is performed regarding pressure ulcers and nutritional status.
3. The patient is provided with carbohydrate loading two hours before the scheduled surgery, compression stockings, antithrombotic medicine (Dalteparin 5000 IU), and a urinary catheter.
4. Patients are encouraged to intake clear glucose-rich fluids until 2 hours before the initiation of anesthesia.

**Perioperative interventions**

1. A nasogastric tube is inserted, and the patient is provided with prophylactic intravenous antibiotics (Piperacillin/Tazobactam 4 g+0.5g and Metronidazole 1.5 g) 30 minutes before the surgical procedure is initiated.
2. A supplemental dose of prophylactic antibiotics (Piperacillin and Metronidazole) is administered in case of fecal contamination.
3. Per- and postoperative screening with Apfel Score is performed to prevent postoperative nausea and vomiting (PONV). In case of an increased risk of postoperative nausea, combination therapy of total intravenous anesthesia, Dexamethasone, and Ondansetron is usually administered. However, dexamethasone is omitted in this study due to a possible influence on the postoperative stress response.
4. “Zero-fluid” balance therapy is targeted with crystalloids (Ringer Acetat, 3 ml/kg/hour).
5. Local infiltration analgesia in the form of Bupivacaine, 2.5 mg (SAD®), is used for the surgical incisions.
6. The nasogastric tube is removed before the cessation of anesthesia.

**Postoperative interventions**

1. The patient is mobilized whilst supervised by a physiotherapist to obtain a faster mobilization.
2. Postoperative analgesic: Paracetamol 1g x 4, Oxycontin 5-10 mg x 2, and Oxynorm 5-10 mg x 2. The postoperative pain is monitored using the VAS (visual analog scale).
3. Enteral nutrition is recommended four hours after the surgical procedure. The patient is motivated to consume oral protein supplementation and medical chewing gum to stimulate bowel motility.
4. The patient is provided with a positive expiratory pressure (PEP) device to prevent the formation of atelectasis and supervised by a physiotherapist.
5. The fluid balance is monitored using a liquid scheme to avoid electrolyte imbalance and obtain a “zero-balance.”
6. Patients are provided with laxatives (Magnesia 1 g x 2)
7. The urinary catheter is discontinued as early as possible after patients have been adequately mobilized.
